# Prospects for NK-based immunotherapy of chronic HBV infection

**DOI:** 10.3389/fimmu.2022.1084109

**Published:** 2022-12-15

**Authors:** Xiaomeng Jin, Jiacheng Bi

**Affiliations:** CAS Key Laboratory of Quantitative Engineering Biology, Shenzhen Institute of Synthetic Biology, Shenzhen Institute of Advanced Technology, Chinese Academy of Sciences, Shenzhen, China

**Keywords:** CHB, CAR-NK, genomic editing, NK cell exhaustion, immune checkpoint

## Abstract

Effective and long-term treatment is required for controlling chronic Hepatitis B Virus (HBV) infection. Natural killer (NK) cells are antiviral innate lymphocytes and represent an essential arm of current immunotherapy. In chronic HBV (CHB), NK cells display altered changes in phenotypes and functions, but preserve antiviral activity, especially for cytolytic activity. On the other hand, NK cells might also cause liver injury in the disease. NK -based immunotherapy, including adoptive NK cell therapy and NK -based checkpoint inhibition, could potentially exploit the antiviral aspect of NK cells for controlling CHB infection while preventing liver tissue damage. Here, we review recent progress in NK cell biology under the context of CHB infection, and discuss potential NK -based immunotherapy strategies for the disease.

## 1 Introduction

### 1.1 An overview of immune response in CHB infection

Nearly 300 million people around the world have chronic hepatitis B infection (1), which might gradually develop into later-stage liver diseases like liver fibrosis, cirrhosis, and cancer. Therefore, CHB represents a key stage for medical interventions to prevent subsequent development of severe liver diseases. The Hepatitis B virus consists of the core protein (HBcAg, nucleocapsid), hepatitis B e antigen (HBeAg), hepatitis B surface antigens (HBsAg, including the large, medium, and small envelope proteins), and the X protein (HBx). Host DNA repair mechanisms convert incoming HBV double-stranded DNA into covalently closed cyclic DNA (cccDNA), which are viral persistence reservoirs and a key obstacle to the complete cure of chronic hepatitis B (2, 3). CHB infection is classified into four clinical phases according to serum HBV DNA load, alanine aminotransferase (ALT) levels, and HBeAg status: the HBeAg-positive immune tolerant (IT) and immune active (IA) phases, as well as the HBeAg-negative inactive carrier (IC) and hepatitis phases (ENEG) (4). Hepatitis B virus (HBV) replicates in hepatocytes, but it’s not cytopathic by itself. HBV associated liver damage is due to anti-HBV immune response in the liver (5). Persistent CHB infection is the consequence of interactions between HBV and the host’s innate and adaptive immune systems.

When HBV has entered the host cells, pattern recognition receptors (PPRs), like Toll-like receptors (TLRs), RIG-I-like receptors (RLRs), and NOD-like receptors (NLRs), recognize pathogen-associated molecular patterns (PAMPs) from HBV, resulting in activation of innate immune cells (e.g., NK cells, macrophages, dendritic cells, monocyte), stimulation of signaling pathway (e.g., Janus kinase-signal transducer and activator of the transcription signaling pathway, NF-κB signaling pathway), and production of chemokine, proinflammatory cytokines and interferons (IFNs), together to form innate defenses against HBV (6, 7). Innate immune response is vital for the early control of HBV infection, as well as for generating HBV-specific adaptive immune response (8). CD8^+^ T cells are the crucial cellular effectors contributing to HBV clearance from the liver with the help of NK cells (9–11). CD8^+^ T cells and B cells encounter HBV antigens presented by antigen-presenting cells (APCs), such as Kupffer cells (KCs), dendritic cells (DCs), and liver sinusoidal endothelial cells (LSECs), leading to the generation of HBV-specific CD8^+^ T cells and anti-HBV antibodies -producing B cells (12, 13). On the other hand, CD4^+^ T cells play a crucial role in facilitating CD8^+^ T cell activation and immune memory responses during HBV infection (14).

However, HBV usually escapes innate and adaptive immune surveillance and establishes persistent infection. HBV reduces the expression of Toll-like receptor 3 (TLR3), melanoma differentiation-associated protein 5 (MDA-5), and retinoic acid-inducible gene I (RIG-I) in DCs and hepatocytes, which reduces responsiveness to PAMPs from HBV and impairs IFN-I production (15). In addition, HBV infection led to reduced expression of TLR2 on hepatocytes in the liver of HBeAg-positive CHB patients, which is associated with a functional reduction in cytokine production (16). HBV also escapes immune recognition by RNA adenosine deaminase ADAR1-dependent viral RNA editing in hepatocytes (17). Furthermore, CHB infection causes functional exhaustion of T cells. For example, HBcAg increases PD-1 expression on CD4^+^ T cells and disturbs the function of CD4^+^ T cells through the ERK, JNK, and PI3K/AKT signaling pathways (18). CHB -associated CD8^+^ T cells are also exhausted and reduced secretion of IL-12,IFN-γ, and TNF-α, as well as cytotoxic activity (19). Moreover, HBV infection led to increased expression of the proapoptotic molecule Bcl-2-like protein 11 in HBV-specific CD8^+^ T cells, possibly leading to CD8^+^ T cell clonal depletion during CHB infection (20). Finally, the humoral immune response is impaired in CHB, as evidenced by fewer HBsAg-specific B cells in CHB patients (21). In addition, Kupffer cells in HBV -carrier mice also mediate humoral immune tolerance by IL-10 production (22). Together these studies demonstrated the compromised anti-HBV immune response in CHB.

## 2 An overview of NK cell biology

NK cells play vital roles in virus control by cytotoxic activity and production of effector cytokines, not only for acute infections but also for chronic infections (23). NK cells activation is determined by the signaling balance between activating receptors and inhibitory receptors. Such balance is usually dysregulated in chronic infections, leading to the dysfunction of NK cells in these contexts (23, 24).

### 2.1 NK cell subsets

NK cells are heterogeneous. In mouse, liver NK cells contain two distinct subsets, the CD49a^-^CD49b^+^ conventional NK cells (cNK) and CD49a^+^CD49b^-^ liver resident NK cells (lrNK) (25). Each accounts for about half of total NK cells in the liver. cNK circulates in the blood, whereas lrNK mainly resides in the hepatic sinusoids (26). Compared with cNK, lrNK display distinct phenotypes, such as expression of CXCR6 and TRAIL. Besides, cNK and lrNK originate from distinct progenitors. cNK originate in the bone marrow, while lrNK are generated from Lin^-^CD122^+^CD49a^+^ progenitors in the fetal liver (27). Similarly, human NK cells include the liver resident NK cell subset. However, their phenotypes and transcription factor requirements are different from those of mouse NK cells. In humans, lrNK could be identified by CXCR6^+^CD49E^-^ markers (28, 29).

### 2.2 Functions of NK cells

NK cells recognize “stressed” cells, such as infected or tumor cells, without major histocompatibility complex restriction or prior sensitization for cytotoxicity and cytokine secretion. NK cells can recognize and display cytolytic activity against virally infected cells by release of lytic granules, or by inducting apoptosis of infected cells by death receptors (30). In addition to cytolytic activity, NK cells are major producers of interferon-γ (IFN-γ) early in the innate immune response (31), as well as producers of tumor necrosis factor-α (TNF-α), interleukin (IL)-10, granulocyte macrophage colony-stimulating factor (GM-CSF), granulocyte colony-stimulating factor (G-CSF) (31). Furthermore, NK cells secrete chemokines, such as lymphotactin (XCL1) and C-X-C motif chemokine ligand 8 (CXCL8, IL-8) (32), which is key to their colocalization with other hematopoietic cells in areas of inflammation (33).

NK cells mediate their antiviral effects through the release of cytokines (e.g., IFN-γ, TNF-α), secretion of cytolytic granules for lysis of infected cells, and induction of target cell apoptosis through crosslinking of cell surface death receptors (e.g., FasL, TRAIL) (34). Among NK cell subsets, lrNK play an antiviral role in the early stage of host infection (35). lrNK produce less IFN-γ than cNK in human and in mice (36, 37), but lrNK produce IFN-γ more rapidly than cNK in response to murine cytomegalovirus (MCMV) infection (35). In addition, lrNK secrete more TNF-α and GM-CSF than cNK following *in vitro* stimulation (37). Murine lrNK expressed higher levels of TRAIL and FasL than cNK, but expressed lower levels of granzyme B (GzmB) and perforin (37). In addition to NK –intrinsic functions, NK cells can also cross-talk with dendritic cells (DC) by cytotoxicity against immature dendritic cells and by interacting with DCs *via* proinflammatory cytokines and chemokines (32, 33). Tissue-resident XCR1^+^ conventional dendritic cells (cDC1) can crosstalk with lrNK to limit early viral load (35).

### 2.3 Mechanisms of NK cell activation

NK cells express the germline-encoded inhibitory receptor, which recognizes “self” -expressing major histocompatibility complex (MHC) class І. NK cells also express activating receptors that recognize “stressed” cells, such as infected or tumor cells. In addition, NK cells express cytokine receptors to type I interferons (IFN-α and IFN-β), interleukin-15 (IL-15), IL-21, IL-18, and IL-12 for activation, as well as receptors to immunosuppressive factors, such as transforming growth factor-β (TGF-β) and IL-10.

The activation of NK cells is determined by the balance between activating signals and inhibitory signals input from cell surface receptors. NK cell activating receptors include natural cytotoxicity receptors (NCR, e.g., NKp30, NKp44, and NKp46), lectin-like receptors (e.g., NKG2D and NKG2C), IgG Fc receptors, and SLAM family receptors (e.g.,2B4, CRACC, and NTB-A) (38). NCRs are immunoglobulin-like type I transmembrane glycoproteins and mediated NK cell activation by the immunoreceptor-based activation motif (ITAM) motif. NKG2D is expressed on NK cells, γδ T cells, and CD8^+^ T cells. Human NKG2D interacts with DAP10 for NK cell activation (39). NK cells respond to “stressed” cells not only directly *via* activating receptors of stressed-induced ligands but also *via* the activating Fc receptor CD16, which recognizes a variety of targets containing antigens that IgG can bind. The initiation of NK-cell effector functions through CD16 depends on transmembrane signaling adaptors, such as FcϵRIγ (also known as FcRγ) and CD3ζ expressed by mature NK cells (40). SLAM-related receptors include 2B4, NTB-A, and CRACC. Src-family kinases phosphorylate SLAM family receptors in the immunoreceptor tyrosine-based switch motifs (ITSM) (41), which is required for further association with SAP-related adapters family. SAP is crucial for human NTB-A and 2B4-mediated NK cell activation (42). However, SLAM family receptors can also negatively regulate NK cell functions (42). In SAP-deficient human NK cells, 2B4 and, possibly, NTB-A turned into inhibitory receptors (43).

In addition to cell surface receptors, NK cell activation is also stimulated by cytokines such as type I IFN, IL-12, IL-15, and IL-18, which are usually produced in response to virus infection (44, 45).Moreover, the interaction with accessory cells also modulates NK cell functions. For example, DC -derived IL-12 promotes both IFN-γ; production and cytotoxicity of NK cells (46).

## 3 NK cells in CHB infection

### 3.1 Antiviral role of NK cells in CHB infection

NK cells represent a major immune cell type in the liver, making up to 50% of hepatic lymphocytes and to an even higher frequency in the livers of patients with CHB infection (47–49). NK cells are essential for controlling CHB infection not only by direct antiviral functions but also by orchestration of adaptive immunity.

#### 3.1.1 Direct antiviral roles

In CHB infection, NK cells contribute to viral control mainly in the early phase of viral infection (23, 33, 50), when NK cell antiviral activity can be directly activated by IFN-α (5). Moreover, the liver of immune-activated (IA) patients contained increased amounts of IL-12, IL-15, and IL-18 (51), which could also contribute to NK cell antiviral activity. Among effector functions of NK cells, cytolytic activity is preferentially increased in CHB infection (49). NK cells display cytolytic activity against an HBV-positive tumor cell line *via* NKp46, whose expression negatively correlates with HBV DNA levels, suggesting that NK cell activity should directly contribute to control HBV replication (52). On the other hand, NK cell-dependent immune response induced by blockade of Qa-1 -NKG2A interaction in an HBV carrier mouse model facilitated viral clearance (53). Poly I:C triggered an NK cell -dependent decrease of serum HBV level and led to HBV clearance in mice (54). After HBV infection, liver NK cells increased expression of cytotoxicity-associated molecules, such as degranulation and tumor necrosis factor (TNF)-related apoptosis-inducing ligand (TRAIL) (55), formed immune synapses with HBV-infected cells to deliver the lytic-granule contents (56), and induced TRAIL-mediated apoptosis of HBV-infected hepatocytes in HBeAg-negative CHB patients during acute liver injury (5).

#### 3.1.2 Immune regulatory roles

Another antiviral aspect for NK cells in CHB is the immune regulation of other cell types (34). NK cells, especially DX5^+^CD49a^−^ conventional NK cells, promote the antiviral activity of CD8^+^ T cells by IFN-γ production in a mouse HBV infection model (10). However, NK cells might paradoxically limit HBV-specific adaptive immunity by killing HBV-specific CD8^+^T cells (57).

### 3.2 Changes of NK cells in CHB

Although NK cells possess anti-HBV potentials, CHB infection induces NK cells into an exhaustion-like status with reduced NK cell percentages in the peripheral blood (58), with dominating expression of inhibitory cell surface receptors, and with decreased effector functions, underlying the dysfunctional host immune responses to HBV in these patients (24, 59).

#### 3.2.1 Phenotypic changes

During CHB, NK cells tend to express higher inhibitory receptors and to downregulate activation receptors (**Table 1**). Inhibitory receptors such as NKG2A, ILT2, and Tim-3 were expressed at high levels, while activating receptors CD16 and NKp30 were downregulated on NK cells (53, 60, 62, 66). HBeAg stimulates IL-10 production in Treg, which elevates expression of NKG2A on NK cells and maintains the frequency of NKG2A^+^Ly49^−^ NK cells on intrahepatic NK cells in CHB (64, 65). CHB infection also increases the levels of the inhibitory receptor Ig-like transcript 2 (ILT2, also named LILRB1, CD85j, LIR-1) on circulating CD56^dim^CD16^+^ NK cells (66). Increased levels of ILT2 mediated by TGF-β1was associated with higher levels of apoptosis in the CD56^dim^CD16^+^ NK cell subset in CHB patients (66). Moreover, Tim-3 expression was increased in CHB -associated peripheral NK cells (62). Similarly, Inhibitory receptor KLRG1^+^ NK cells were increased in the blood and liver of HBsAg –positive CHB patients (63).

**Table 1:** Alterations of NK cells in CHB.

	Receptor	Alteration in CHB	Reference
Activating Receptor	NKp30	↓	(60)
	NKG2C	↑	(49)
	NKG2D	↓(IT)	(61)
	CD16	↓	(58)
	CD244 (2B4)	↓(IT)	(61)
Inhibitory Receptor	Tim-3	↑	(62)
	KLRG1	↑	(63)
	CD94 / NKG2A	↑	(64, 65)
	ILT2	↑(IT, IA, ENEG)	(66)
Cytokine Receptor	CD122	↓(ASC)	(67)
Effector molecule	IFN-γ	↓	(68)
	TNF-ɑ	↓	(68)

IT, HBeAg-positive immune tolerant phase; IA, HBeAg-positive immune active phase; ENEG, HBeAg-negative hepatitis phase; ASC, asymptomatic chronic HBV carriers.

On the other hand, CD122 (the β chain of IL-2 receptor (IL-2R) and CD25 (the α chain of IL-2R), and CD132 (the common γ chain) were decreased on CD56^dim^ NK cells, possibly leading to NK cell dysfunction in asymptomatic CHB carriers (67), who’s in the immunosuppressive phase with normal serum alanine transaminase (ALT) levels despite large antigens and high levels of HBV replication (69). Moreover, the frequency of NKG2D^+^ NK cells from peripheral blood mononuclear cells (PBMC) and liver was decreased in CHB carriers (IT, immune tolerance) (70).

#### 3.2.2 Functional changes

Phenotypic changes underlie the dysfunctional status of NK cells in CHB, possibly as a result of the overall dysregulated immune status in the disease. For example, cNK from CHB patients reduced IFN-γ and TNF-α production, although their cytotoxicity is increased (49, 55). Such defect is associated with decreased CD122 on CD56^dim^ NK cells in asymptomatic CHB carriers. In line with this, IL-15 and/or IL-2, combined with IFN-α2, could reverse the dysfunctional status of CD56^dim^ NK cells through the CD122-mediated signaling pathway, accompanied by the increase of p-STAT5 (67). Moreover, IL-15-mediated AKT/mTOR pathway activation is impaired in NK cells from CHB patients, suggesting that NK cells of CHB patients may have metabolic defects (58). High levels of inhibitory cytokines production in the liver in CHB, such as IL-10, might contribute to the inhibition of IFN-γ production (68).

#### 3.2.3 Other changes

In CHB, NK cells presented transcriptional similarity with exhausted T cells, e.g., increased expression of TOX (Thymocyte selection associated high mobility group BOX) or NR4A (Nuclear Receptor subfamily 4 group A)-family transcription factors and their immune checkpoint targets (58). In addition, T-bet, the transcription factor critical for NK cell functional maturation, was significantly down-regulated in NK cells from CHB, which is in consistent with their overall dysfunctional status.

### 3.3 Pathogenic role of NK cells in CHB infection

NK cells not only control virus replication in the liver, but also could cause liver injury when NK cells become over activated in some contexts (5, 71, 72). Activated NK cells aggravate hepatic inflammation and stimulate hepatocyte death by secretion of inflammatory cytokines and by TRAIL-mediated apoptosis of hepatocytes in chronic hepatitis B patients (73, 74). NK cells also produced IFN-γ and TNF-α to exacerbate liver injury (74), and increased Fas ligand (FasL) or NKG2D ligand expression on hepatocytes to promote apoptosis (70, 75). Therefore, although NK cells contribute to virus control, they might also be pathogenic in CHB depending on the context.

## 4 Potential NK-based immunotherapy for CHB infection

Currently, two antiviral drugs are available for treating CHB infection: nucleus(t)ide analogs (NUCs) and interferons (IFNs) (76). NUCs suppress HBV replication and contribute to prevent HBV-associated end-stage liver disease, but usually fail to clear HBsAg in these patients (77), suggesting that most CHB patients might require an uncertain or even lifelong period of NUC treatment (77, 78). On the other hand, Peg-IFN-α treatment results in HBeAg clearance and HBsAg seroconversion in some patients. However, Peg-IFN-α treatment has shortcomings, such as poor tolerability, serious and frequent side effects, and the manner of subcutaneous administration (79, 80).Current therapies require long-term medication and seldom achieve functional cures for chronic infections, as defined by serum clearance of hepatitis B surface antigen (HBsAg) and the virus DNA. Therefore, developing new therapeutics with improved safety and efficacy is necessary.

**Figure 1:**
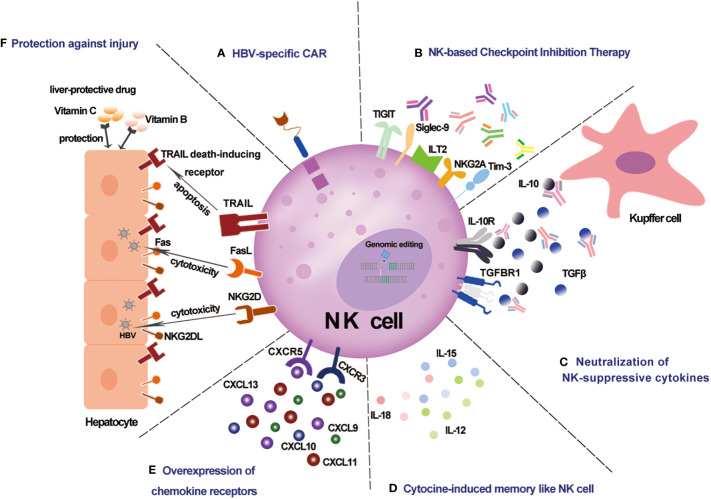
Schematic representation of potential NK-based immunotherapy for CHB infection. **(A)** HBV-specific CAR NK cells specifically recognize HBV-infected cells. **(B)** NK -based checkpoint inhibition therapy against NK cell functional exhaustion. **(C)** Neutralization of NK-suppressive cytokines against the immunosuppressive chronic inflammatory microenvironment. **(D)** Cytokine-induced memorylike NK cells with enhanced anti-HBV potency. **(E)** Overexpression of chemokine receptors enhances liver -trafficking. **(F)** Protection against injury by liver protective drugs for NK -based therapy.

NK cells play a crucial role in host antiviral immunity. NK cells are not only protective in the HBV mouse model (10), but might also help to control HBV by potential NK -based immunotherapy, including NK cell therapy, checkpoint inhibition therapy, and others, which have proved safe and effective against cancers (81, 82) (**Figure 1**).

## 4.1 NK cell therapy

Adoptive transfer of NK cells exploits the effector functions of ex vivo expanded NK cells (83), from NK cell line, cord blood, peripheral blood, or induced pluripotent stem cells (iPSCs). Such strategy has shown safety (84, 85) and therapeutic potential for the control of various viruses, not only in mouse models, but also in clinical trials. Adoptive transfer of mouse NK cells provides resistance to MCMV (86), and adoptive transfer of MCMV -specific Ly49H-expressing NK cells rendered even greater resistance (87). Adoptive NK cell therapy for HIV control in AIDS patients has been performed in clinical trials to determine safety, tolerability, alloreactivity, and efficacy (NCT03346499, NCT03899480). In addition, a Phase I/II escalating-dose clinical trial of NK cells adoptive immunotherapy for control of COVID-19 has been conducted (NCT04578210). These studies indicated that adoptive NK cell therapy is safe, and offers viral control efficacy for a wide range of viruses, possibly also for CHB.

In addition to the intrinsic antiviral potential of NK cells, genetic engineering of NK cells could further enhance adoptively transferred NK cell potency against CHB by promoting recognition of HBV, prolonging persistence, enhancing activity, overcoming exhaustion, and increasing migration to the liver. Chimeric antigen receptors (CAR)-NK cells exploit the antigen-targeting capabilities of CAR (88). The safety and efficacy of CAR-NK cell therapy has been shown in anti-tumor therapy (89), suggesting that CAR-NK cell therapy should be an ideal platform for disease treatment, possibly also for control of virus infection (89). For an anti-HBV CAR design, HBV -specific CAR consists of single-chain Ab fragment scFv specifically recognizing HBV components, a hinge domain, a transmembrane domain, and signaling domain(s). For example, CD8^+^ T cells were armed with a CAR specifically recognizing the S domain of HBV envelope proteins (S, M, and L protein, combined as HBsAg), which could signal through intracellular CD28 and CD3ζ domain. These HBV –specific CAR-T cells efficiently control HBV replication in immunocompetent HBV transgenic mice (90). Similarly HBsAg-CAR T cells (HBs-G4m-CAR T cells) in HBV-infected human liver chimeric mice cause a reduction of serum HBsAg and HBV DNA levels (91). Based on these studies on T cells, NK cells could also be armed with HBV -specific CAR in adoptive cell therapy against CHB to test its virus control efficacy.

In addition to CAR modifications, gene editing for disruption of checkpoint molecules that suppress NK cell activity is also an important strategy. For instance, AKT/mTOR signaling pathway was impaired in CHB -associated circulating NK cells (58). Disruption of *CISH* gene expression in primary or iPSC -derived NK cells metabolically reprogrammed NK cells to improve *in vivo* persistence and enhanced effector functions (92, 93), accompanied by increased IL-15-mediated JAK-STAT signaling activity (93), suggesting that *CISH* deletion might preserve the AKT/mTOR signaling in NK cells for CHB therapy. Furthermore, inhibitory cell surface immune checkpoints mediate the inhibitory signaling inputs in the chronic inflammatory microenvironment of CHB. NKG2A blockade has been shown to promote HBV clearance in a mouse model (53), and blocking NKG2A *in vitro* enhances the cytotoxicity of NK cells from active CHB patients’ peripheral blood. These suggest that targeting the *KLRC1* gene might benefit adoptively transferred NK cell therapy for active CHB patients.

For adoptive NK cell therapy against CHB, the migration of NK cells to the liver is an essential topic. Conventional NK cells do not display liver-homing preference as liver -resident NK cells do (25). While large-scale production of liver -resident NK cells has not been reported, the distribution also remains unclear of the adoptively transferred NK cells produced from common sources, such as peripheral blood, cord blood, or iPSCs. Alternatively, forced expression of chemokine receptors in NK cells might facilitate NK cell migration to the liver. CXCR2 -overexpressing NK cells have been shown to exhibit improved trafficking to the tumor site (94), suggesting that similar strategies of ectopic expression of chemokine receptors in NK cells should be feasible. Serum CXCL9, CXCL10, CXCL11, and CXCL13 levels are increased in CHB patients (95, 96), suggesting that their receptors (CXCR3 for CXCL9/10/11 and CXCR5 for CXCL13) should be ideal targets in CHB. In line with this, CD8^+^T cells expressing C-X-C motif chemokine receptor 5 (CXCR5), recruited by high levels of C-X-C motif chemokine ligand 13 (CXCL13) in CHB patients, favors viral control in CHB infection (97). Therefore, it would be interesting for future studies to determine the benefits of CXCR5 -overexpressing adoptively transferred NK cells for treating CHB.

Although NK cells are innate lymphocytes, recent studies indicate that they also possess adaptive features. Vaccination against HBV induced a CD56^dim^CD57^+^ CD69^+^ KLRG1^+^ NK cell subset which displayed higher activity upon HBsAg stimulation compared with NK cells from unvaccinated subjects (98). Moreover, cytokine-induced memory-like (CIML) NK cells, generated by short-term activation with a cytokine combination of IL-12, IL-15, and IL-18, displayed enhanced proliferation and persistence *in vivo* (99). The enhanced capabilities and the simple generation protocol make it applicable, maybe also for virus control. Adoptive transfer of CIML-NK cells for tumor control has shown promising results in various tumor models (99, 100), suggesting that HBV –specific memory NK cells or CIML-NK cells could be adoptively transferred for treating CHB as well.

### 4.2 NK-based checkpoint inhibition therapy

The activity of NK cells is overall inhibited during CHB infection (101). Immune-checkpoint inhibitors, whose therapeutic value has been revealed in the field of cancer therapy, could potentially relieve suppression of NK cells’ activity also in CHB by preventing inhibitory signaling through inhibitory checkpoint receptors. Blockades of inhibitory receptors like NKG2A, Tim-3, and Siglec-9 have been shown to promote NK cell function in CHB infection (53, 62, 102) (**Table 2**). For example, NKG2A^+^ NK cell is dysfunctional in HBeAg-positive CHB patients (64). Blocking NKG2A interaction with HLA-E increased *in vitro* cytolytic activity of peripheral NK cells from patients with active CHB (53). Similarly, Tim-3 expression is remarkably increased in circulating NK cells from CHB patients (62). *In vitro* cytotoxicity by PBMCs or NK cells from CHB patients was enhanced in the presence of Tim-3 blockers (62). Moreover, inhibitory receptor Siglec-9 is commonly expressed on CD3^−^CD56^dim^ NK cells. Blocking Siglec-9 on NK cells among PBMCs from HBsAg-positive CHB patients significantly improved the production of IFN-γ, TNF-α, and CD107a of NK cells (102), indicating that blocking Siglec-9 may restore the dysregulation of NK cells in HBsAg-positive CHB patients. Therefore, the blockade of immune checkpoint signaling may relieve the negative regulation of NK cells and revive the antiviral function of NK cells for CHB control.

**Table 2:** NK cell checkpoint inhibition for CHB.

Checkpoint	Influence on NK cell functions after blockade	Reference
NKG2A	cytotoxicity (↑)	(53)
Tim-3	cytotoxicity (↑)	(62)
Siglec-9	IFN-γ (↑) TNF-ɑ (↑) CD107a (↑)	(102)

### 4.3 Neutralization of NK-suppressive cytokines

Immunosuppressive cytokines contribute to NK cell receptor imbalance and NK cell dysfunction during CHB infection. TGF-β1 reduced the expression level of NKG2D/DAP10 and 2B4/SAP in patients in the immune tolerance (IT) phase of CHB infection, whose NK cells displayed impaired function (61). Neutralization of TGF-β1 restored the level of NKG2D and 2B4 on NK cells *in vitro* (61). Neutralizing IL-10 restored IFN-γ production by CD56^bright^ and CD56^dim^ NK cells in patients with active CHB infection (68), whereas in CHB patients without IL-10 elevation or noticeable liver inflammation, only blockade of both IL-10 and TGF-β restored NK function (68). Modulation of the cytokine milieu has shown potentials in cancer treatment (103), which might also be applied for treating CHB. Therefore, targeting immunosuppressive cytokines represents an intervention strategy to boost NK cell-based immunotherapy for CHB infection.

### 4.4 Protection against injury

Protection from potential liver injury is also important for designing anti-HBV NK -based immunotherapy. Blocking chemokines responsive to γ–2/IFN-γ inducible protein 10/C-X-C motif chemokine ligand 10(CRG-2/IP-10/CXCL10) and monokine induced by interferon-γ/C-X-C motif chemokine ligand 9 (Mig/CXCL9) *in vivo* relieves liver injury while preserving the antiviral potential of HBV–specific cytotoxic T lymphocytes (96), suggesting that these are targets for liver protection in immunotherapy for CHB. Furthermore, combing NK-based immunotherapy with liver -protective drugs might prevent NK cell -mediated liver injury while preserving its antiviral functions. Vitamin C consumption attenuated liver injury of CCl4-treated mice by normalizing metabolism and relieving inflammatory responses in the liver (104), while Vitamin C is also an antioxidant that may stimulate or at least maintain the activation of NK cells (105, 106). Vitamin B is a conventional liver-protective drug, and it has been used clinically for the treatment of chronic hepatitis B (107), which, like vitamin C, could also promote the activation of NK cells (108).

## 5 Conclusion and prospect

Currently, effective and tolerable treatment for CHB is required. With the rapid progress in NK-based immunotherapy for cancers, attention has also been paid to exploiting the antiviral potential of NK cells for virus control, especially those for chronic infection. In recent years, our knowledge of the beneficial role of NK cells in CHB has been expanding. Although the activity of NK cells is usually dysregulated in CHB, NK cells play important roles in controlling HBV during acute and chronic infection by direct antiviral effects and immunomodulatory function. NK -based immunotherapy has been shown safe in treating cancers and is under investigation in clinical trials for virus control. Despite the anti-HBV potentials, dysregulated activity of NK cells in CHB might also lead to liver injury. Therefore, future design of NK -based immunotherapy for CHB should pay attention to preventing non-specific tissue injury while preserving anti-HBV effector functions of NK cells.

Taken together, NK -based immunotherapy has shown potentials in cancer therapy, which also draws our attention to exploiting its potent antiviral ability. However, limited studies have investigated antiviral potentials of NK -based immunotherapy, especially for CHB. Future studies should, on one hand, focus on technical improvements in the design for potent, precise, and persistent NK -based therapies and, on the other hand, keep on unveiling the complex interactions between HBV and NK cells. Hopefully, future progress in these two aspects could lead to novel therapeutic opportunities for CHB.

## Author contributions

XJ and JB conceived and wrote the manuscript. All authors contributed to the article and approved the submitted version.
